# Longitudinal association between toenail zinc levels and the incidence of diabetes among American young adults: The CARDIA Trace Element Study

**DOI:** 10.1038/srep23155

**Published:** 2016-03-16

**Authors:** Jong Suk Park, Pengcheng Xun, Jing Li, Steve J. Morris, David R. Jacobs, Kiang Liu, Ka He

**Affiliations:** 1 Department of Epidemiology and Biostatistics, School of Public Health–Bloomington, Indiana University, Bloomington, Indiana, USA; 2 Department of Endocrinology and Metabolism, Yonsei University College of Medicine, Seoul, Republic of Korea; 3 The Research Reactor Center, University of Missouri-Columbia and Harry S. Truman Memorial Veterans Hospital, Columbia, Missouri, USA; 4 Division of Epidemiology and Community Health, School of Public Health, University of Minnesota, Minneapolis, Minnesota, USA; 5 Department of Preventive Medicine, Feinberg School of Medicine, Northwestern University, Chicago, Illinois, USA

## Abstract

Data on primary prevention of zinc status and diabetes risk are sparse and inconsistent. Of note, the previous studies measured either dietary zinc intake with questionnaire or zinc status in serum or hair. Toenail zinc levels are reliable biomarkers of a relatively long-term exposure. A total of 3,960 American young adults, aged 20–32 years, free of diabetes at baseline in 1987 when toenail clippings were collected, were examined for incident diabetes through 2010. Toenail zinc levels were measured with an inductively-coupled-plasma mass spectroscopy method. Incident diabetes cases were identified by fasting or non-fasting plasma glucose levels, oral glucose tolerance tests, hemoglobin A1C levels, and/or antidiabetic medications. During the 23-year follow-up, 418 incident diabetes occurred. After adjusted for age, sex, ethnicity, study center, body mass index, education, smoking status, alcohol consumption, physical activity, family history of diabetes, homeostasis model assessment of insulin resistance, and other dietary and non-dietary potential confounders, the hazard ratio of incident diabetes comparing the highest to the lowest quartile of toenail zinc levels was 1.21 (95% CI: 0.90–1.63; *P*_trend_ = 0.20). Findings from this study do not support the hypothesis that zinc status is inversely and longitudinally associated with the incidence of diabetes in American young adults.

The prevalence of diabetes mellitus is increasing rapidly all over the world and has become a major public health concern. Many factors including diet have been recognized to be associated with the risk of diabetes. Zinc is an essential micronutrient, which is important for cellular growth and maintenance of biological processes. Zinc levels in pancreatic islet cells are tightly regulated, so that dysregulation of zinc metabolism could affect the synthesis and secretion of insulin as well as glycemic control^1^,^2^,^3^. Thus, it has been hypothesized that zinc status is inversely related to diabetes risk.

Previous studies investigated zinc status in diabetic patients and findings were controversial. Some studies showed no difference in zinc levels between diabetic patients and apparently healthy controls^4^,^5^, while other studies reported that diabetes was strongly associated with zinc deficiency^3^,^6^,^7^,^8^. Data on primary prevention of zinc status and diabetes risk are sparse and inconsistent. Two cohort studies reported that dietary zinc intake is associated with lower risk of type 2 diabetes^9^,^10^. Recently, another cohort study found that higher serum zinc was associated with higher risk of type 2 diabetes^11^. However, another cohort study conducted in a Chinese population found no association between dietary zinc intake and hyperglycemia^12^. To date, one Cochrane review based on three intervention studies found that zinc supplementation was not related to incidence of diabetes^13^.

Of note, the previous studies either measured dietary zinc intake with questionnaire or zinc status in serum or hair. Because of the large variation, questionnaire may not be able to capture zinc intake. Also, the exposure time frame reflected by serum zinc is relatively short^14^. Human hair has been used as a biomarker of zinc status in the studies of diabetes^15^,^16^. However, hair analysis is prone to be affected by cosmetic procedures such as dyeing, bleaching and permanent waving that alter trace element content in hair^17^. In fact, hair as a biomarker for assessing trace element status has been questioned^18^,^19^,^20^. By contrast, toenail zinc levels are reliable biomarkers of a relatively long-term exposure and have been used in other studies^21^,^22^,^23^. In addition, study has indicated a good reproducibility of toenail zinc level over a 6-year period^24^.

Therefore, we prospectively investigated toenail zinc levels in relation to the incidence of diabetes in a large cohort of American young adults using data from the Coronary Artery Risk Development in Young Adults (CARDIA) Study.

## Methods

The CARDIA study is a multi-center, prospective study to investigate risk factors for cardiovascular disease in American young adults. The detailed information on the study design and protocol has been published^25^. Briefly, 5,114 African American and Caucasian men and women, aged 18–30 years, were enrolled from 4 US cities (Birmingham, AL; Chicago, IL; Minneapolis, MN and Oakland, CA) from 1985 to 1986. Seven follow-up examinations have been conducted in 1987/88, 1990/91, 1992/93, 1995/96, 2000/01, 2005/06, and 2010/11. About 68.4% (3,499/5,114) of participants in the original cohort returned in 2010/11 examination, which resulted in an average follow-up rate of 94.6% between two adjacent visits. The study design, data collection, and analyses were approved by the institutional review boards at all the participating institutions: the University of Alabama at Birmingham, the University of Minnesota, Northwestern University, and Kaiser Permanente, and written informed consent was obtained from all participants. And all the methods were carried out in accordance with the approved guidelines.

### Study population

Study population is 4,362 CARDIA participants who provided toenail clippings in 1987/88. At baseline, we excluded participants who had diabetes (*n* = 11); participants with missing data on important covariates (*n* = 182) [including glucose (*n* = 66), alcohol consumption (*n* = 1), body mass index (BMI) (*n* = 2), magnesium and long chain n-3 polyunsaturated fatty acid (LCn-3PUFAs) intake (*n* = 98), and family history of diabetes (*n* = 15)]; those who had insufficient information for defining incident diabetes during the follow-up (*n* = 3); and women who were pregnant at any examination (*n* = 206). After these exclusions, a total of 3,960 participants remained in the analysis.

### Assessment of toenail zinc

Toenail clippings were collected with a stainless-steel clipper from all 10 toes by the participants themselves during the clinical examination in 1987 and stored. All toenail clippings were processed with a washing procedure in a sonicator with deionized water. Toenail zinc levels were measured by the Inductively-Coupled-Plasma Mass Spectroscopy method at the University of Missouri Research Reactor^26^. Toenail samples were analyzed in random order by the laboratory personnel blinded to other clinical measures. The coefficient of variation in duplicated subsamples for the toenail zinc measure was 6.5% in the present study. A study showed that Spearman correlation coefficient for the reproducibility of toenail zinc over 6 years was 0.58^24^.

### Assessment of glucose, insulin, HOMA-IR, OGTT, and HbA1c

Fasting plasma glucose and insulin levels were determined by the hexokinase ultraviolet method and radioimmunoassay, respectively, at exam years 0, 7, 10, 15, 20, and 25. To assure comparability of plasma glucose and insulin across examinations, they were recalibrated, which was described in detail previously^27^. Homeostasis model assessment of insulin resistance (HOMA-IR) was calculated as glucose (mmol/L) × insulin (mU/L)/22.5. Two-hour plasma glucose levels were measured from a standard 2-hour oral glucose tolerance test (OGTT) at years 10, 20 and 25. Glycated hemoglobin (HbA1c) was assessed using a Tosoh G7 high performance liquid chromatography instrument (Tosoh Bioscience) at years 20 and 25. The inter-assay CVs were 2.0–3.0%.

### Ascertainment of diabetes

At any follow-up examination, participants with one or more of the followings were determined as having incident diabetes: 1) fasting plasma glucose ≥7.0 mmol/L; 2) non-fasting plasma glucose ≥11.1 mmol/L; 3) postprandial 2-hour plasma glucose ≥11.1 mmol/L from an OGTT; 4) HbA1c ≥6.5% (≥48 mmol/mol); or 5) reported use of antidiabetic medications, which were verified by medication names^28^.

We could not clearly distinguish diabetes type, because some participants were young at diagnosis and used insulin as treatment. Therefore, we used the term “diabetes” rather than “type 2 diabetes”, but the great majority of cases were type 2 diabetes.

### Measurement of other covariates

Age, gender, ethnicity, education level, smoking status, and alcohol consumption were self-reported, obtained by interview or self-administered questionnaire. Smoking status was classified into three groups: never, former, and current smokers. Alcohol consumption was classified into four groups according to total daily intake: never, 0.1–9.9, 10–19.9, and ≥20 g/day. Body weight and height were directly measured in light clothes without shoes, BMI was calculated as weight (kg) divided by height squared (m^2^). Physical activity was assessed using the CARDIA Physical Activity History Questionnaire, an interviewer-based self-report of frequency of participation in each of 13 categories of sports and exercise over the previous 12 months. A score of 100 exercise units (EU) is approximately equivalent to participation in vigorous activity for 2–3 hours/week during 6 months of the year^29^,^30^. Family history of diabetes was defined as either mother or father having diabetes.

### Assessment of zinc intake and other dietary factors

Briefly, we collected dietary data, including zinc intake and assessed three times at baseline, exam years 7 and 20, using a validated interviewer-administered CARDIA Diet History Questionnaire. Information on supplement use was also collected. Zinc intake represented the sum of dietary zinc intake and zinc supplementation. The details of dietary assessment and validation in CARDIA have been described previously^31^,^32^.

### Statistical analysis

Distributions of baseline characteristics categorized by quartiles of toenail zinc levels were described by mean (standard deviations), median (inter-quartile ranges), or percentage, whichever was proper. The difference across categories of toenail zinc concentration was compared using analysis of variance, Kruskal-Wallis test, or chi-squared test as appropriate.

We calculated the incidence of diabetes according to the quartiles of toenail zinc levels. Participants contributed to person-time from the baseline to the time when a case was identified, a participant was censored, or the end of the study, whichever came first. Cox proportional hazards models were used to estimate the hazard ratios (HRs) and 95% confidence intervals (CIs) of the incident diabetes. Owing to the limited literature on this research topic, potential confounders were identified mainly based on statistical tests and our previous knowledge in studying zinc in relation to other health end points. We considered several sequential models in the main analysis: The initial analysis (model 1) was adjusted for age, sex, ethnicity, study center, BMI and baseline HOMA-IR. In model 2, we further adjusted for education, smoking status, alcohol consumption, physical activity, and family history of diabetes. In model 3, we additionally adjusted for intakes of LCn-3PUFAs and magnesium, and toenail levels of selenium, mercury and chromium. Continuous variables were created using the median values in each quartile for trend tests.

In addition, we explored possible interactions between toenail zinc concentrations and pre-specified potential effect modifiers by adding corresponding multiplicative interaction terms in the models, followed by the likelihood ratio test. If the interaction was statistically significant, we reported stratified results. Moreover, we did several sensitivity analyses to test the robustness of our results. First, we substituted waist circumference for BMI in the models. Second, because the cutoff point of fasting glucose for defining diabetes was changed from 140–126 mg/dL in 1997, we used a cutoff point of 140 mg/dL at exam years before 1997. Third, considering diabetes may also cause the change in zinc metabolism, we excluded the baseline HOMA-IR from the model.

All analyses were carried out by using SAS 9.4 (SAS Institute, Inc., Cary, NC, USA). All tests were two sided and *P* ≤ 0.05 was considered statistically significant.

## Results

A total of 3,960 participants were divided into four groups according to their toenail zinc concentrations. Baseline clinical and biochemical characteristics of the study population were presented in Table 1. The median values of toenail zinc across quartiles were 31.2, 49.6, 61.1, and 77.5 μg/g. Compared with participants in the lowest quartile of toenail zinc concentrations, those in the highest quartile were slightly older and more likely to be Caucasians, exercised more, drank more, had a higher education level, and higher levels of fasting glucose, toenail selenium, mercury and chromium, and lower levels of fasting insulin. In addition, participants with higher toenail zinc levels consumed more magnesium.

During the 23 years of follow-up, 418 incident cases of diabetes were identified, including 265 cases determined by fasting glucose criteria and 3, 48, 62 and 40 cases determined by non-fasting glucose, 2-hour glucose after OGTT, HbA1c and anti-diabetic medication use criteria, respectively.

A non-significant association was found between toenail zinc levels and the incidence of diabetes after adjustment for demographic and major lifestyle variables, including BMI (Table 2). As compared with participants in the lowest quartile of toenail zinc, the fully adjusted hazard ratios of incident diabetes from the second to the fourth quartiles were 1.14 (95% CI: 0.85, 1.52), 1.17 (95% CI: 0.87, 1.59), and 1.21 (95% CI: 0.90, 1.63), respectively (*P* for linear trend = 0.20).

We further explored the association between dietary zinc intake and diabetes risk. Similarly, no significant association was observed (Appendix Table). After multivariable adjustment, the hazard ratios of incident diabetes across quartiles of dietary zinc intake were 1.00 (reference), 1.03 (95% CI: 0.76, 1.39), 0.97 (95% CI: 0.67, 1.42), and 1.27 (95% CI: 0.81, 2.01), respectively (*P* for linear trend = 0.23). Since two previous studies reported an inverse association between dietary zinc intake and incidence of diabetes^9^,^10^, we pooled the results from these two studies with our findings. The combined hazards ratio of diabetes was 0.85 (95% CI: 0.57, 1.26) compared the highest to the lowest zinc intake group.

Our findings were not materially modified by race, sex and weight status (data not shown). In the sensitivity analyses, the results were not appreciably changed when we substituted waist circumference for BMI in the models. In addition, the findings remained when we used different definitions of diabetes based on the time period of the examination. Moreover, excluding baseline HOMA-IR from the model did not change the results materially, either (data not shown).

## Conclusions

In this large cohort study, we prospectively investigated toenail zinc concentration and dietary zinc intake in relation to the incidence of diabetes among American young adults. No significant associations were observed between toenail zinc or dietary zinc intake and diabetes incidence during 23 years of follow-up.

Previous studies found that serum zinc levels in people with diabetes were lower than that in people without diabetes in the control group^6^,^7^,^8^. The possible explanations were that urinary excretion of zinc might increase and intestinal reabsorption of endogenous zinc might be impaired in people with diabetes^33^,^34^. A systematic review published in 2012 summarized data from 3 studies on type 1 diabetes and 22 studies on type 2 diabetes, and concluded that zinc supplementation has beneficial effects on glycemic control^35^. However, in apparently healthy individuals, no significant reduction in glucose concentration following zinc supplementation was observed^36^,^37^,^38^, which is generally consistent with our findings. In addition, clinical trials have failed to demonstrate that zinc supplementation will reduce the incidence of type 2 diabetes mellitus^12^.

To date, two cohort studies examined the longitudinal association between dietary zinc intake and the incidence of type 2 diabetes^9^,^10^. Using data from the Nurses’ Health Study, Sun *et al.* reported that zinc intake was inversely associated with incidence of type 2 diabetes, though the generalizability may be limited by the fact that the great majority of participants are white nurses^9^. Findings from Sun *et al.* were supported by a study among 8,921 Australian women, aged 45–50 years, with 6 years of follow-up^10^. Nevertheless, when we pooled the results from these two studies with findings in our study, no significant association was found between dietary zinc intake and diabetes risk. Of note, dietary zinc intake quantified by the food frequency questionnaire may not be able to capture the accurate zinc intake due to the relatively large variation of zinc content in food^15^.

In the present study, we do not find any significant association between toenail zinc concentration or dietary zinc intake and the incidence of diabetes. There may be a few possible explanations for the null findings: 1) random measurement errors in both toenail zinc and zinc intake may attenuate any potential association^39^,^40^; 2) although the advantage of toenails as a long-term exposure biomarker of zinc status has been recognized^15^,^24^, one toenail zinc measurement at baseline may not reflect the changes in zinc status during the follow-up. Since the changes are likely to be non-differential, the possible association may be attenuated; 3) comparing to previous studies^9^,^10^,^11^, average zinc intake in the present study was substantially higher. For example, the average dietary zinc intake were 9.4 mg/day (median) in the Nurses’ Health Study^9^, and 10.66 mg/day (mean) in the Australian Longitudinal Study on Women’s Health^10^, while mean dietary zinc intake in our study was 16.7 mg/day. As a result, participants even in the lowest zinc group may have already obtained the maximal benefit, further increase in zinc status or intake may not provide additional benefit; and 4) a negative confounder, i.e. a factor with an opposite association with zinc status and incidence of diabetes, could bias any possible association towards the null. However, the likelihood should be small since we carefully considered a number of potential confounding factors in the analyses^41^.

The strengths of our study include a long-term prospective study design, a large cohort of young adults, and well balanced for gender and ethnicity. The previous cohort studies were conducted in Caucasian women or Asian population. Our study adds additional information on men and African American. Also, both zinc intake and toenail zinc concentration were measured and the results were consistent. In addition, our diabetes cases were not self-reported, but defined mainly based on fasting and non-fasting glucose levels, postprandial glucose levels from an OGTT and HbA1c measurements besides antidiabetic medications.

Our study also has limitations. First, the possibility of confounding from unknown or unmeasured factors cannot be completely excluded. Second, toenail zinc concentrations were only measured once at baseline. Thus, we were unable to study any change in zinc levels in relation to the incidence of diabetes. For example, if participants with low zinc levels at baseline increased zinc intakes later on, any possible association would be attenuated. Finally, the young participants in the present study were recruited from urban areas, the generalizability may be limited. However, the CARDIA sampling frame insures desired population balance at baseline, and thus the population can be considered representative of the baseline city populations.

In conclusion, in this cohort of American young adults, we did not find any significant longitudinal association between toenail zinc levels or dietary zinc intake and the incidence of diabetes. Our results do not support the hypothesis that zinc may contribute to the reduction of diabetes risk.

## Additional Information

**How to cite this article**: Park, J. S. *et al.* Longitudinal association between toenail zinc levels and the incidence of diabetes among American young adults: The CARDIA Trace Element Study. *Sci. Rep.* **6**, 23155; doi: 10.1038/srep23155 (2016).

## Supplementary Material

Supplementary Appendix table

## Figures and Tables

**Table 1:** Baseline characteristics by quartiles of toenail zinc levels, the CARDIA Trace Element Study, 1987–2010 (*n* = 3,960)*.

Characteristics	Quartiles of toenail zinc levels				Total	*P* value†
	Q1(lowest)	Q2	Q3	Q4 (highest)		
*n*	991	994	986	989	3,960	–
Toenail zinc (μg/g)	31.20 (22.40–37.70)	49.60 (46.60–52.40)	61.10 (58.50–64.40)	77.50 (71.40–87.10)	55.50 (42.80–67.50)	–
Toenail selenium (μg/g)	0.80 (0.72–0.90)	0.83 (0.75–0.92)	0.86 (0.77–0.95)	0.86 (0.78–0.96)	0.84 (0.75–0.94)	<0.01
Toenail mercury (μg/g)	0.19 (0.11–0.32)	0.20 (0.12–0.36)	0.23 (0.14–0.40)	0.24 (0.13–0.41)	0.21 (0.12–0.37)	<0.01
Toenail chromium (μg/g)	0.47 (0.21–1.01)	0.55 (0.25–1.22)	0.54 (0.28–1.15)	0.63 (0.34–1.43)	0.55 (0.26–1.21)	<0.01
Age (year)	26.60 ± 3.70	26.96 ± 3.57	27.28 ± 3.53	27.29 ± 3.61	27.03 ± 3.61	<0.01
Female (%)	54.39	49.30	51.42	53.69	52.20	0.09
African American (%)	56.91	51.51	43.81	39.23	47.88	<0.01
Current smoker (%)	19.48	21.83	21.10	21.54	20.98	0.58
Physical activity score (EU)	288 (140–510)	336 (179–541)	328 (179–532)	327 (190–556)	322 (168–535)	<0.01
Education (year)	14.06 ± 2.19	14.16 ± 2.32	14.29 ± 2.46	14.44 ± 3.63	14.24 ± 2.72	0.01
BMI (kg/m^2^)	25.07 ± 5.64	25.15 ± 5.29	25.10 ± 5.10	25.45 ± 5.52	25.19 ± 5.39	0.36
FH of diabetes (%)	29.57	27.57	26.27	27.91	27.83	0.44
Insulin (μU/mL)	10.88 ± 7.55	11.22 ± 9.13	10.30 ± 7.91	10.37 ± 7.06	10.69 ± 7.96	0.03
Glucose (mg/dL)	81.09 ± 8.60	81.89 ± 8.50	82.52 ± 8.12	81.78 ± 7.88	81.82 ± 8.30	<0.01
HOMA-IR (μU/mL · mg/dL)	1.56 ± 1.15	1.62 ± 1.39	1.50 ± 1.25	1.50 ± 1.08	1.55 ± 1.22	0.07
Energy intake (kcal/day)	2798.51 ± 1349.55	2896.95 ± 1357.13	2861.47 ± 1337.46	2752.53 ± 1275.45	2827.41 ± 1330.99	0.07
Alcohol intake (ml/day)	3.39 (0.00–13.20)	5.07 (0.00–16.23)	6.09 (0.00–16.23)	5.41 (0.00–16.93)	5.07 (0.00–15.90)	<0.01
LCn-3PUFA intake (g/day)	0.12 ± 0.24	0.11 ± 0.14	0.12 ± 0.17	0.12 ± 0.15	0.12 ± 0.18	0.82
Magnesium intake (mg/day)	361.55 ± 179.65	395.19 ± 194.78	406.91 ± 214.33	402.61 ± 206.57	391.57 ± 199.95	<0.01

Abbreviations: BMI: body mass index; CARDIA: Coronary Artery Risk Development in Young Adults; EU: exercise unit; FH: family history; HOMA-IR: homeostatic model assessment–insulin resistance; LCn-3PUFA: long chain omega-3 polyunsaturated fatty acid.

^*^Data are medians (inter-quartile ranges), means ± standard deviations or proportions.

^†^P values are for any difference across the quartiles of toenail zinc levels by using analysis of variance, Kruskal-Wallis test, or chi-squared test as appropriate.

**Table 2:** Multivariable-adjusted HRs and 95% CIs for incidence of diabetes by quartiles of toenail zinc levels, the CARDIA Trace Element Study, 1987–2010 (*n* = 3,960)*.

	Quartile of toenail zinc levels				*P* for linear trend†
	Q1 (lowest)	Q2	Q3	Q4 (highest)	
Toenail zinc (μg/g)	<42.9	42.9–55.4	55.5–67.5	>67.6	
No. of participants	991	994	986	989	
No. of events	107	103	99	109	
Model 1‡	1.00	1.10 (0.83, 1.46)	1.13 (0.84, 1.52)	1.22 (0.91, 1.62)	0.18
Model 2§	1.00	1.13 (0.85, 1.50)	1.15 (0.86, 1.56)	1.21 (0.90, 1.62)	0.20
Model 3||	1.00	1.14 (0.85, 1.52)	1.17 (0.87, 1.59)	1.21 (0.90, 1.63)	0.20

Abbreviations: BMI: body mass index; CI: confidence interval; HOMA-IR: homeostatic model assessment – insulin resistance; HR: hazard ratio; LCn-3PUFA: long chain omega-3 polyunsaturated fatty acid.

^*^All models were constructed by using Cox proportional hazards model.

^†^Medians of zinc in each quartile were used for testing the linear trend.

^‡^Model 1: adjustment for age (continuous), gender, ethnicity (African American or Caucasian), study center, BMI (continuous) and baseline HOMA-IR (quartiles).

^§^Model 2: model 1 with additional adjustment for education (continuous), smoking status (never smokers, former smokers, or current smokers), alcohol consumption (0, 0.1–9.9, 10.0–19.9 or ≥20 g/day), physical activity (quartiles) and family history of diabetes (yes or no).

^||^Model 3: model 2 with additional adjustment for intakes (quartiles) of LCn-3PUFAs and magnesium, and toenail elements (quartiles) including selenium, mercury and chromium.
