# Supplementary material for: Longitudinal association between toenail zinc levels and the incidence of diabetes among American young adults: The CARDIA Trace Element Study
Source: Sci Rep. 2016 Mar 16;6:23155. doi: 10.1038/srep23155 (PMC4793256; doi:10.1038/srep23155)
Supplement: Supplementary Appendix table [file srep23155-s1.doc]

**Longitudinal association between toenail zinc levels and incidence of diabetes among American young adults: The CARDIA Trace Element Study**

Running title: toenail zinc and incidence of diabetes

Jong Suk Park MD, PhD ^1,2^; Pengcheng Xun, MD, PhD^1^; Jing Li, MS^1^; Steve J Morris, PhD^3^; David R Jacobs Jr., PhD^4^; Kiang Liu, PhD^5^; and Ka He, MD, ScD^1,*^

1. Department of Epidemiology and Biostatistics, School of Public Health--Bloomington, Indiana University, Bloomington, Indiana, USA
2. Department of Endocrinology and Metabolism, Yonsei University College of Medicine, Seoul, Republic of Korea
3. The Research Reactor Center, University of Missouri-Columbia and Harry S. Truman Memorial Veterans Hospital, Columbia, Missouri, USA
4. Division of Epidemiology and Community Health, School of Public Health, University of Minnesota, Minneapolis, Minnesota, USA
5. Department of Preventive Medicine, Feinberg School of Medicine, Northwestern University, Chicago, Illinois, USA

***Corresponding author:** Dr. Ka He, Department of Epidemiology and Biostatistics, School of Public Health--Bloomington, Indiana University, 1025 E. Seventh Street, HP C032, Bloomington, Indiana 47405, USA; Email: kahe@indiana.edu; Tel: +1-812-855-7977

Word count: 2,781 (abstract: 200)

Table: 2 /Appendix table: 1

**Appendix Table**- Multivariable-adjusted HRs and 95% CIs of incidence of diabetes by quartiles of dietary zinc intake, the CARDIA Trace Element Study, 1985-2010 (*n*=4,555) **^*^**

|  | Quartile of dietary zinc levels | | | | *P* for  linear trend^†^ |
| --- | --- | --- | --- | --- | --- |
|  | Q1 (lowest) | Q2 | Q3 | Q4 (highest) |  |
| Zinc intake (mg/day) | <12.15 | 12.15-16.74 | 16.75-23.34 | >23.34 |  |
| No. of participants | 1,138 | 1,140 | 1,138 | 1,139 |  |
| No. of events | 136 | 124 | 108 | 116 |  |
| Model 1‡ | 1.00 | 0.89 (0.69, 1.13) | 0.85 (0.65, 1.10) | 0.98 (0.75, 1.27) | 0.97 |
| Model 2^§^ | 1.00 | 0.93 (0.73, 1.19) | 0.90 (0.69, 1.18) | 1.05 (0.80, 1.38) | 0.60 |
| Model 3|| | 1.00 | 1.03 (0.76, 1.39) | 0.97 (0.67, 1.42) | 1.27 (0.81, 2.01) | 0.23 |

Abbreviations: BMI: body mass index; CARDIA: Coronary Artery Risk Development in Young Adults; CI: confidence interval; HOMA-IR: homeostatic model assessment - insulin resistance; HR: hazard ratio; LCn-3PUFA: long chain omega-3 polyunsaturated fatty acid.

^*^ All models were constructed by using Cox proportional hazards regression analysis.

^†^ Medians of zinc in each quartile were used for testing the linear trend.

^‡^ Model 1: adjustment for age (continuous), gender, ethnicity (African American or Caucasian), study center, BMI (continuous) and baseline HOMA-IR (quartiles).

^§^ Model 2: model 1 with additional adjustment for education (continuous), smoking status (never smokers, former smokers, or current smokers), alcohol consumption (0, 0.1-9.9, 10.0-19.9 or ≥20 g/day), physical activity (quartiles) and family history of diabetes (yes or no).

^||^ Model 3: model 2 with additional adjustment for intakes (quartiles) of LCn-3PUFAs, magnesium, iron and total energy.
